# Dietary intervention of mice using an improved Multiple Artificial-gravity Research System (MARS) under artificial 1 *g*

**DOI:** 10.1038/s41526-019-0077-0

**Published:** 2019-07-08

**Authors:** Chie Matsuda, Tamotsu Kato, Sayo Inoue-Suzuki, Jun Kikuchi, Toshiko Ohta, Masaharu Kagawa, Masahira Hattori, Hiroe Kobayashi, Dai Shiba, Masaki Shirakawa, Hiroyasu Mizuno, Satoshi Furukawa, Chiaki Mukai, Hiroshi Ohno

**Affiliations:** 1 Space Biomedical Research Group, Human Spaceflight Technology Directorate, JAXA, Tsukuba, Japan; 2 Laboratory for Intestinal Ecosystem, RIKEN Center for Integrative Medical Sciences (IMS), Yokohama, Japan; 3 Graduate School of Medical Life Science, Yokohama City University, Yokohama, Japan; 4 Institute of Nutrition Sciences, Kagawa Nutrition University, Saitama, Japan; 5 Environmental Metabolic Analysis Research Team, RIKEN Center for Sustainable Resource Science, Yokohama, Japan; 6 Graduate School of Advanced Science and Engineering, Waseda University, Tokyo, Japan; 7 JEM Utilization Center, Human Spaceflight Technology Directorate, JAXA, Tsukuba, Japan

**Keywords:** Biological sciences, Health care

## Abstract

Japan Aerospace Exploration Agency (JAXA) has developed mouse habitat cage units equipped with an artificial gravity-producing centrifuge, called the Multiple Artificial-gravity Research System (MARS), that enables single housing of a mouse under artificial gravity (AG) in orbit. This is a report on a hardware evaluation. The MARS underwent improvement in water leakage under microgravity (MG), and was used in the second JAXA mouse mission to evaluate the effect of AG and diet on mouse biological system simultaneously. Twelve mice were divided into four groups of three, with each group fed a diet either with or without fructo-oligosaccharide and housed singly either at 1 *g* AG or MG for 30 days on the International Space Station, then safely returned to the Earth. Body weight tended to increase in AG mice and decrease in MG mice after spaceflight, but these differences were not significant. This indicates that the improved MARS may be useful in evaluating AG and dietary intervention for space flown mice.

## Introduction

The Multiple Artificial-gravity Research System (MARS) developed by the Japan Aerospace Exploration Agency (JAXA) is a fully-equipped mouse environment for spaceflight with an artificial gravity-producing centrifuge. It comprises mouse habitat cage units (HCU), a transportation cage unit (TCU), and a centrifuge-equipped biological experiment facility (CBEF).^1^ On its first mission, HCUs were found to have developed water leakage under microgravity (MG).^1^ Since this leakage may have adversely affected the health of experimental mice, an improvement of MARS was attempted in order to retrieve space flown mice back on the Earth at healthy state.

Following this, a second mission was conducted in order to evaluate the efficacy of the prebiotics fructo-oligosaccharide (FOS), which has been previously demonstrated on the ground to support gut microbiome balance in mice,^2^ as well as strength and bone density in rats.^3^ We evaluated changes in body weight before and after spaceflight in response to FOS content in the diet, as well as in response to artificial gravity (AG) versus MG using the improved MARS,^1^ and manuscripts on effect of FOS on gut microbiome and bone health during the spaceflight are in preparation.

## Results and discussion

### Improvement of MARS hardware

**Fig. 1:**
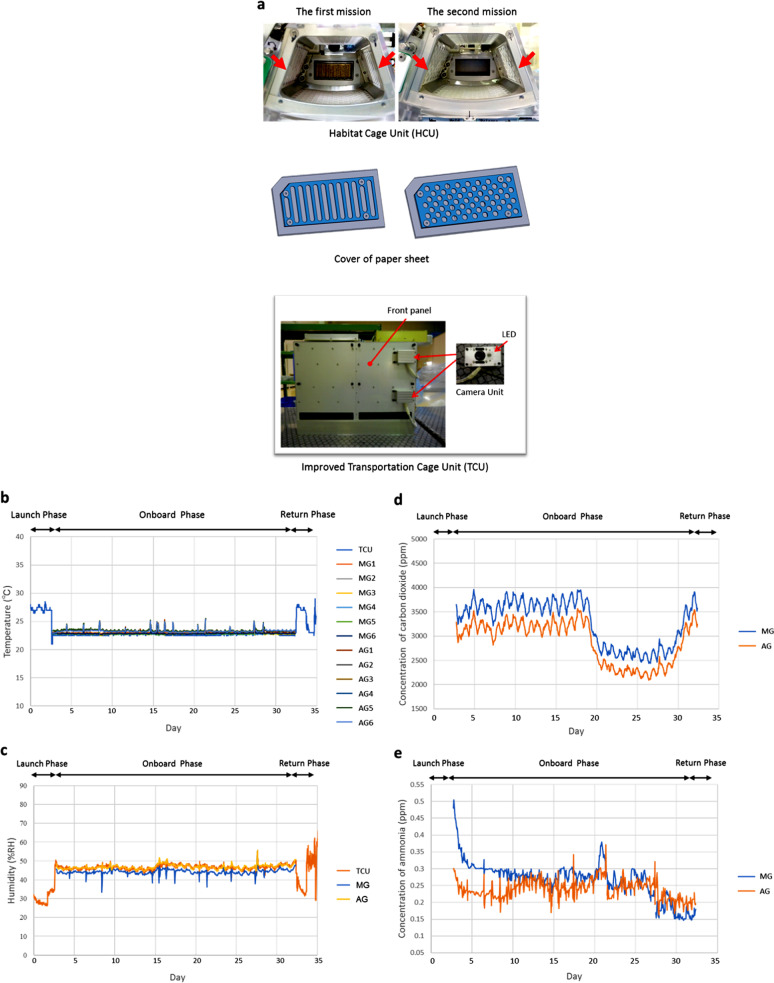
Hardware and environmental measurements for the second JAXA mouse mission **a** Upper: a habitat cage unit (HCU) used in the first (left) and the second (right) JAXA mouse missions. Red arrows indicate the paper sheet covers. Middle: design of the paper sheet covers was modified to protect from tearing by mice. Lower; an improved transportation cage unit (TCU). **b**–**e** Environmental data. Temperature **b** and humidity **c** in the TCU and the HCU were recorded by data loggers. **b** Changes in temperature in the TCU and each cage of the HCU during the mission are shown. **c** changes in humidity in the TCU (orange) and microgravity (MG, in yellow) and artificial 1 *g* gravity (AG, in blue) sections of the HCU during the mission are shown. Gas concentration of carbon dioxide **d** and ammonia **e** in the Centrifuge-equipped Biological Experiment Facility (CBEF) were monitored as described previously^1^ and those in the MG *(*blue) and the AG (yellow) sections of the CBEF during the mission are shown

During the first mission, water leakage from the watering nozzle in the mouse HCU was observed. The failed HCU was retrieved from the International Space Station (ISS) and the problem was investigated on the ground. The absorbent paper sheet on the HCU inner wall, intended to absorb urine from the mice, was torn into debris by mice, and interfered with the water nozzle.^1^ In order to address these issues, the following changes were made to the HCU to prevent future leakage. First, a slit on the cover of the paper sheet was modified from oblong to a small circle (Fig. 1a), reducing the ability of mice to tear the sheet. Second, the original water nozzle included a handle for mice to grip in order to facilitate drinking easily in an MG environment. However, mice were able to drink without the handle during the first mission, and they were removed for the second mission to reduce leakage risks. After these modifications, the modified HCU (named the “HCU rev.1”) showed no leakage during the second mission. The modifications also improved the ability to recover fecal pellets.

In addition, several modifications to the TCU were enacted for the second mission. First, it was equipped with video camera systems to enable monitoring of the mice during transportation (Fig. 1a). Second, the cages of TCU were replaced with a cartridge-type cassette of six concatenated cages instead of individual separate cage units. This made handling of the hardware easier and faster, resulting in savings in mission costs and precious crew time.

Although mice are social animals, a single housing was chosen for HCU to ensure each individual fecal sampling. In order to reduce socially related stress of mouse from single housing,^4^ a window was installed on a side wall of an HCU and allow mice to see their neighboring mouse during the habitation. The use of HCU, however, may not be necessarily be suitable for behavioral or psychological experiments as single housing might influence social activities of a mouse.

### Flight experiment

Twelve 9-week-old mice were selected for spaceflight based on body weight and water and food consumption, and flown to the ISS by SpaceX-12. On board, they were housed singly for 30 days under an AG or MG environment. Acclimation of mice for spaceflight were performed in three phases as shown in Supplementary Fig. 1a. Mice were fed a modified AIN-93G diet either with (+) or without (−) FOS during acclimation with a flight-type food bar^1^ (phase III in Supplementary Fig. 1a) and orbital housing. The modified AIN-93G without FOS contained 5% cellulose instead of 5% FOS, and was energy equivalent to the modified AIN-93G with FOS. Ingredients of the modified diets with or without FOS were identical except cellulose and FOS, which were indigestible. Half of the mice were fed with FOS to examine its impact on gut microbiome,^5^ and loss of disused muscle and bone^1^ during spaceflight. The mice housed under AG showed lethargic behavior for 2 days after centrifugal start on the first day of orbital housing. This has been previously reported for mice during adaptation to hyper-gravity environments created by centrifuge on the Earth.^6^ All mice in an AG environment adapted within 5 days of launch (L+5), with the exception of Mouse AG3 (where AG or MG plus a number represents each mouse under AG or MG conditions), which was assumed slow adaptation to AG. Intake of water in AG3 had not been confirmed by 7 days after launch (L + 7), and therefore water gel was supplied to this individual. Outside of this, food and water intake of all mice was normal during orbit. MG mice were housed for 30 days in the HCU under MG, and AG mice were maintained for 30 days under artificial 1 *g*, including 24.55 total hours of weekly maintenance period.

More than five pieces of unflooded fecal pellets from all the mice were collected at every sampling point (Supplementary Fig. 1) including in the ISS and stored at −80 °C. Tegaderm improved the fecal pellet collection protocol in the ISS microgravity environment. The floor material of AG, MG, and ground control were identical that had holes made for urine and feces to pass through. Coprophagia was observed in MG mice as well as those housed in normal cages on the Earth, but AG mice were unable to do so, ase they could not reach accumulating feces under the HCU floor. All mice were single housed to eliminate gastrointestinal effect by coprophagia of cage mates. The presence or absence of recycling of their own feces was different among the housing groups, however, this was considered to be a limitation of space experiment. Development of cage floor to control coprophagia should be considered to study gut microbiome especially for group housing.

### Environmental data during orbital housing

Measurements of temperature and humidity as well as concentration of carbon dioxide and ammonia in MARS demonstrated its suitability for housing mice (Fig. 1b–e).^1^ Although there were rapid rises in humidity and concentration of ammonia in the HCU during its first mission (Supplemental Fig. 3a, b of ref. ^1^), these were relatively stable in the second mission. This was probably owing to the anti-leakage modifications in the HCU revision 1 (rev.1). Environmental data during MHU-1 is reported in Supplemental Figs. 2a, b and 3a, b of ref. ^1^. As airflow was supplied from the AG section to the MG section in one direction, concentration of CO_2_ in the MG was higher than that in the AG section. Similar concentration distribution of CO_2_ in CBEF was observed in the first mission (Supplemental Fig. 3a of ref. ^11^ Radiation dosages in the TCU and the HCU during spaceflight showed that the absorbed dose rate was 0.26 × 0.01 mGy/day and the equivalent dose rate was 0.53 ± 0.04 mSv/day during this experiment, which was slightly higher than in the first mission.^1^ Achievement of a more stable MARS should open new space research possibilities.

### Body weight analysis

**Fig. 2:**
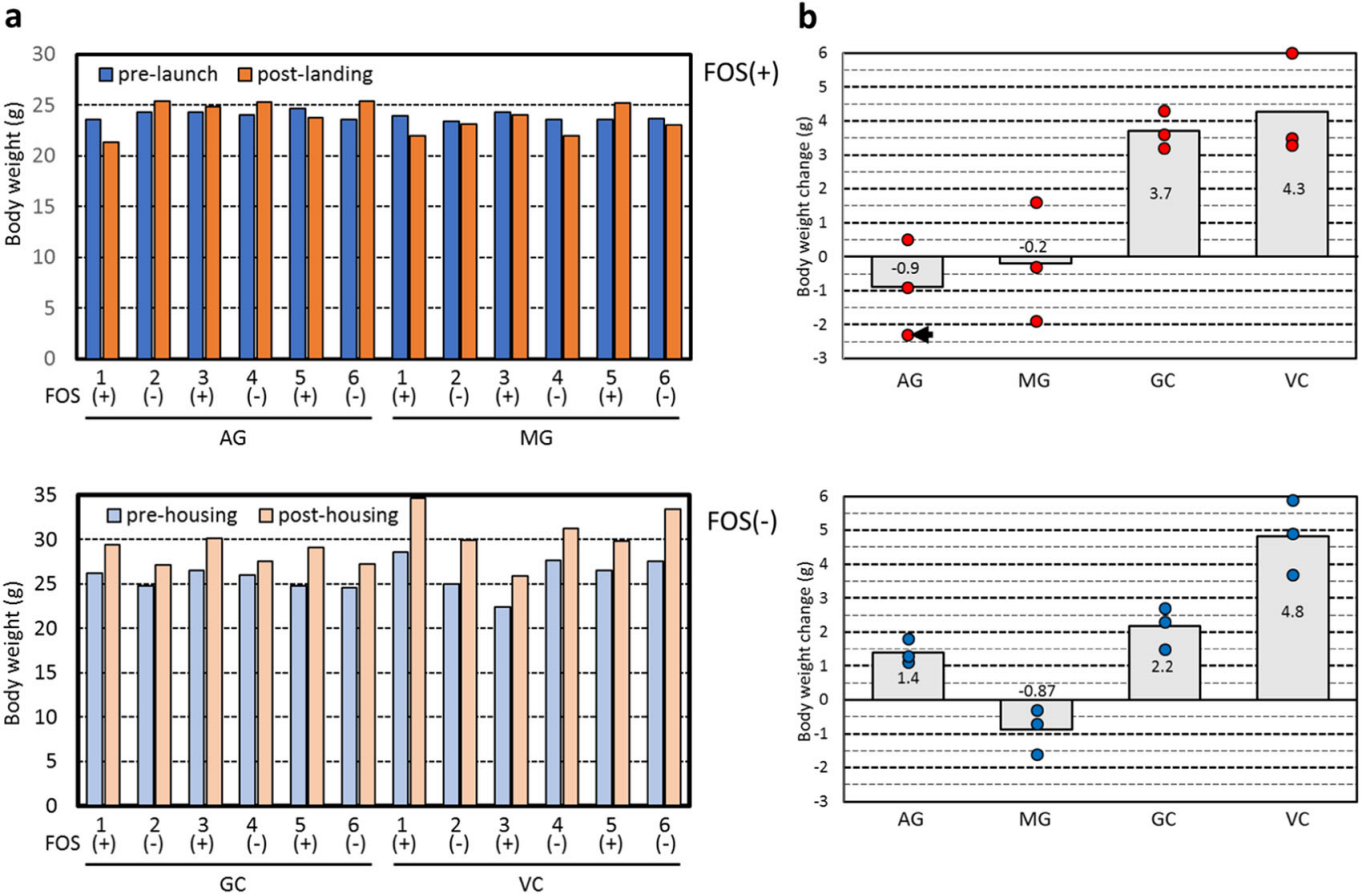
Body weight of mice for spaceflight, ground control, and vivarium control before and after experiments **a** The body weight of mice for spaceflight on the day before launch ranged from 23.6 to 24.7 g. AG indicates that mice were housed under artificial 1 *g* gravity (*n* = 6); MG indicates that mice were housed under microgravity (*n* = 6). GC indicates mice for ground control housed in the ground model of the HCU (*n* = 6); VC indicates mice for vivarium control housed in a standard cage (*n* = 6). **b** Changes in body weight between before and after spaceflight (or the reproductive experiments for the spaceflight for GC and VC mice) were expressed as mean (bars with mean value), and values of each mice (dots) are also shown. Arrow indicates AG1 with water leakage in the TCU

All 12 mice returned alive to the earth. No abnormal health conditions were observed after landing. Changes in body weight were compared with control mice on the ground housed in an HCU model (GC), and mice for vivarium control were housed in standard cage (VC) (Fig. 2a, b). Four of the six AG mice gained weight compared with pre-launch, whereas five of the six MG mice lost weight. Similar changes in body weight were observed in the first mission.^1^ Average body weight change in AG and MG mice in housing without water leakage in the first mission was 2.2 ± 0.5 g and 0.6 ± 0.5 g, respectively,^1^ and three mice in housing with water leakage showed a considerable loss in weight. However, observed body weight changes from the two missions must be compared with care, as mouse age and period of orbital housing were different. Of note, the water tank was empty in the TCU of AG1, whose body weight decreased (Fig. 2a). As described above, we improved HCU to prevent water leakage for the second mission, but further refinement should be enacted to develop the TCU to cope with water leakage in future missions. It is possible that water leakage in the TCU is related to weight loss. However, it was expected that Mouse AG1 could take water, and that the water leakage in the TCU had minimal influence on its health conditions. As no obvious symptoms of dehydration were observed in post-flight AG1, it can be assumed that the water leakage occurred gradually and slowly during the return flight. A comparison of body weight changes in all experimental group (AG, MG, GC, and VC with and without FOS) including AG1 using a Kruskal–Wallis test showed that the changes were statistically significant (*P* = 0.0053); however, arbitrary two group did not show statistical significance according to Dunn’s multiple comparison. As there were no significant differences in body weight changes among the four flight mice groups (AG and MG with/without FOS; *P* = 0.16 with Krustal–Walllis test for non-parametric 450–451) between before and after spaceflight, we suggest that FOS had no impact on body weight changes during spaceflight regardless of AG or MG. Blood collections by tail-cut were conducted, as the technical verification for future experiments, from AG4 and MG6 on L + 17 and from AG3 and MG1 on L + 18, and a manuscript on details of blood collection on board is in preparation.

Based on the success of this mission, the improved MARS can be regarded as a useful single housing system for evaluation of AG, and could be used to analyze biological systems of mice in orbit. During the second mission, a dietary intervention experiment using FOS was conducted and analyzable fecal, blood, tissue, and organ samples were obtained from all mice. To the best of our knowledge, this is the first study to collect feces from individual mice in orbit, enabling the tracing of changes in intestinal environment during spaceflight. Based on our experiment, FOS was shown to have no significant impact on weight between pre- and post-spaceflight regardless of living condition of mice. Analysis of feces, blood, and organ samples as well as impact of FOS on the physiology of the mice returned from the ISS will be reported in future. Recently, DNA sequencing^7^ and amplification^8^ were successfully achieved on the ISS. Analyses of feces or blood samples from mice housed in the improved MARS by these techniques could lead to development of health monitoring method during spaceflight.

## Methods

### Ethical approval

Animal protocols were reviewed and approved by the Institutional Animal Care and Use Committee of JAXA (protocol number; 016-018), Yokohama City University (T-A-15-005), NASA (protocol number; FLT-17-106), and Explora Biolabs (EB15-010). The experimental procedures performed by astronauts were reviewed and approved by JAXA Astronauts Research Review Board (28-1) and NASA Flight Institutional Review Board (2158).

### Environmental data in orbit

Temperature and humidity in the TCU and the HCU were recorded by data loggers (DS1923; KN Laboratories, Inc., Osaka, Japan). Gas concentration of carbon dioxide and ammonia in the CBEF were monitored as described previously.^1^ Radiation dosages in the TCU and the HCU during spaceflight was measured by Bio PADLES (TLD/CR39, Fukuvi Chemical Industry) monitoring devices.^9^

### Mouse diets

For all experiments, modified AIN-93G^10^ (Oriental Yeast Co., Ltd., Tokyo, Japan) with or without 5% FOS by weight (Meiji Food Materia Co. Ltd., Tokyo, Japan) was used in this study. In the modification, concentrations of vitamins and an antioxidant t-butylhydroquinone were increased (Supplementary Table 1) to compensate for the loss of these ingredients by oxidation during prolonged preservation in the ISS before use. A verification experiment using the modified diet confirmed that there were no significant differences in body weight changes, or in food and water consumption by mice during the course of a week. This confirmed that the dietary modification had low impact on eating behavior (Supplementary Fig. 2). On orbit, the launched mice were fed the diets that had been launched before arrival of mice on the ISS, because the diets needed to be set in advance. Nutrient components of the diet after preservation were analyzed to confirm that stored diets would satisfy mouse nutrient requirements^11^ (Supplementary Fig. 2).

### Animals and pre-launch treatment

The overview of pre-launch acclimation conducted in the Space Station Processing Facility Science Annex at Kennedy Space Center (KSC) (FL, USA) is shown in Supplementary Fig. 1a. Five-week old C57BL/6 J mice (stock number 000664) were purchased from The Jackson Laboratory (Bar Harbor, ME, USA) for space and ground control experiments. Distilled water containing iodine at 0.2 mg/L was supplied during acclimation and orbital housing. Specific pathogen-free (SPF) conditions were confirmed by PCR test using body surface swabs and feces at 13 days before launch (Charles River Research Animal Diagnostic Services, MA, USA). Twelve flight mice, including six fed an FOS(+) diet and six fed an FOS(−) diet, were given a health check by the NASA Launch Facility veterinarian before installation in the TCU.

### Mouse housing in orbit

Three of the six mice consuming the FOS(+) diet were assigned to an AG environment at 1 *g* using MARS with centrifugation at 77 rpm^1^: AG1, AG3, and AG5. They were accompanied by three mice consuming the FOS(−) diet: AG2, AG4, and AG6. Three of the six mice consuming the FOS(+) diet were placed in microgravity: MG1, MG3, and MG5, joined by three of the mice consuming the FOS(−) diet: MG2, MG4, and MG6 (Supplementary Fig. 1b).

### Return phase

The Dragon vehicle loading the TCU was splashed down in the Pacific Ocean off the coast of California (Supplementary Fig. 1c). All the mice were killed and dissected within 36.5 hours of splash down.

### Feces collection

Feces collection was conducted using a sterile medical adhesive sheet (Tegaderm, 3 m Japan, Tokyo, Japan) both in orbit and on the ground. Time points of fecal sampling are shown in Supplemental Fig. 1a–c.

### Ground and vivarium control experiments

A ground control experiment was conducted from 18 January to 22 March 2018 at JAXA (Tsukuba, Japan). SPF conditions of the mice were confirmed using PCR 4 days after the receipt of mice (Charles River Japan, Ibaraki, Japan). Environmental conditions of housing were as close as possible to acclimation conditions at KSC. Three of mice fed the FOS(+) diet and three fed the FOS(−) diet as ground controls were housed in the essential model of TCU and the ground model of HCU to mimic the space experiment. For vivarium control, three mice fed the FOS(+) diet and three fed the FOS(−) diet were housed in the same type of cages used for pre-launch acclimation at KSC. Mouse diets and water consumption were the same as used in flight.

### Statistical analysis

Body weight, food consumption, and water consumption data are presented as means ± SE. Dunn’s multiple comparison test and a Kruskal–Wallis test were applied for non-parametric statistical analysis.

### Reporting summary

Further information on research design is available in the Nature Research Reporting Summary linked to this article.

## Supplementary information

Supplementary Figures and Tables.

Supplementary Movie 1.

Supplementary Movie 2.

Supplementary Movie 3.

Supplementary Movie 4.

Supplementary Movie 5.

Supplementary Movie 6.

Supplementary Movie 7.

Supplementary Movie 8.

Reporting summary

## Data Availability

The data that support the findings of this study are available from the corresponding authors upon reasonable request.

## Supplementary information

**Supplementary information** accompanies the paper on the *npj Microgravity* website (10.1038/s41526-019-0077-0).
