# Supplementary material for: Dietary intervention of mice using an improved Multiple Artificial-gravity Research System (MARS) under artificial 1 g
Source: NPJ Microgravity. 2019 Jul 8;5:16. doi: 10.1038/s41526-019-0077-0 (PMC6614370; doi:10.1038/s41526-019-0077-0)
Supplement: Supplementary file 1 — Supplementary Figures and Tables. [file 41526_2019_77_MOESM1_ESM.pdf]

Supplementary Figure 1. Overview of the second JAXA mouse mission. (a) Schedule of pre-launch operation at the Kennedy Space Center. All mice were housed singly to analyze intestinal environment individually at a temperature of 23 ± 3 ° C, humidity of 40 ± 15 % and 12 hours light-dark cycle. The selected mice were installed to the TCU and launched to the ISS via Space-X12 on August 14, 2017 (GMT). (b) Orbital housing. The Space-X12 arrived at the ISS on August 17 2017 (GMT), and mice were relocated from the TCU to the HCU by an astronaut. Housing of mice in orbit was conducted for 30 days. Food replacement and refilling of water were performed weekly. (c) Return phase. The mice were relocated from the HCU to the TCU for return to Earth on September 16, 2017 (GMT), and the Dragon vehicle loading the TCU splashed down in the Pacific Ocean off the coast of California at 14:17 on September 17, 2017 (GMT). Handover of the TCU retrieved by NASA to JAXA was done at 15:57 on the same day (GMT). The TCU was then transported to Explora Biolabs in San Diego by car.

Supplementary Figure 2. Biocompatibility of the modified AIN-93G diet under different preservation conditions. AIN-93G with or without FOS was preserved for 0, 3 and 5 months at 25 ° C or for 1.5 months at 40 ° C after manufacture to examine biocompatibility of preserved diets. Following 2 weeks acclimation with pelleted original AIN-93G[10], C57BL/6J male mice at 6 weeks old purchased from CLEA Japan Inc (Tokyo, Japan) were fed the preserved diets in flight-type bar form, and housed for a week to monitor body weight and food and water consumption. (a) Relative body weight change, (b) diet and (c) water consumptions of the mice fed the modified AIN-93G after various preservation conditions. Data for the diet preserved for 0 (blue), 3 (orange) and 5 months at 25 ° C (gray), and 1.5 months at 40 ° C (yellow) are shown as means ± SE (n = 5).

Supplementary Table 1. Nutritional components and ingredients of modified AIN-93G

Supplementary Table 2. Vitamin content in modified AIN-93G after preservation for 1 year at 25 ° C. Requirement of vitamins for mice are also shown.[11]

Supplementary Videos

Supplementary Movie 1. Movie of AG1 mouse under dark phase during onboard habitation (L+20).

Supplementary Movie 2. Movie of AG2 mouse under dark phase during onboard habitation (L+20).

Supplementary Movie 3. Movie of MG1 mouse under dark phase during onboard habitation (L+20).

Supplementary Movie 4. Movie of MG2 mouse under dark phase during onboard habitation (L+20).

Supplementary Movie 5. Movie of AG1 mouse under light phase during onboard habitation (L+20).

Supplementary Movie 6. Movie of AG2 mouse under light phase during onboard habitation (L+20).

Supplementary Movie 7. Movie of MG1 mouse under light phase during onboard habitation (L+20).

Supplementary Movie 8. Movie of MG2 mouse under light phase during onboard habitation (L+20).

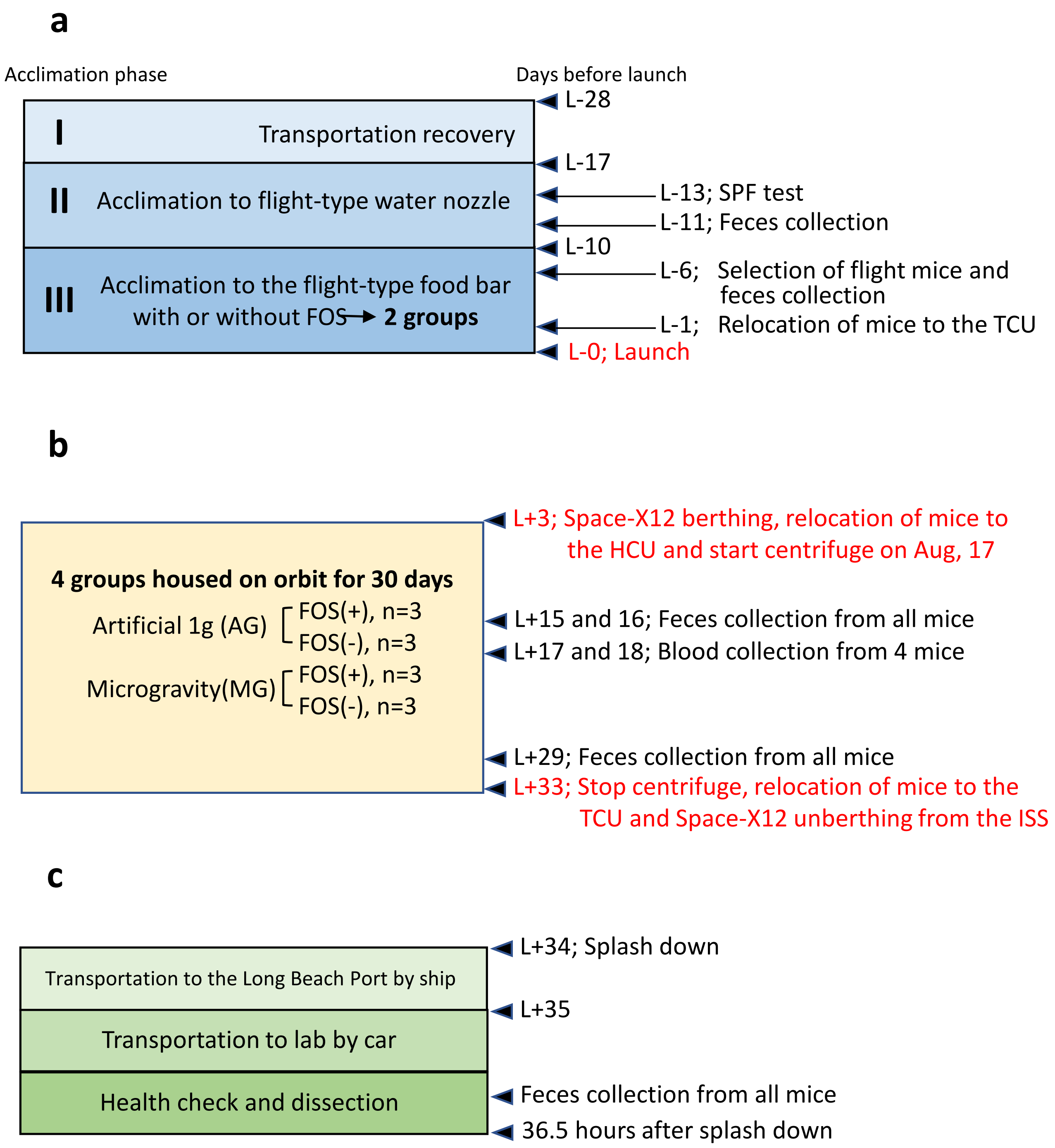

Supplementary Figs. 1

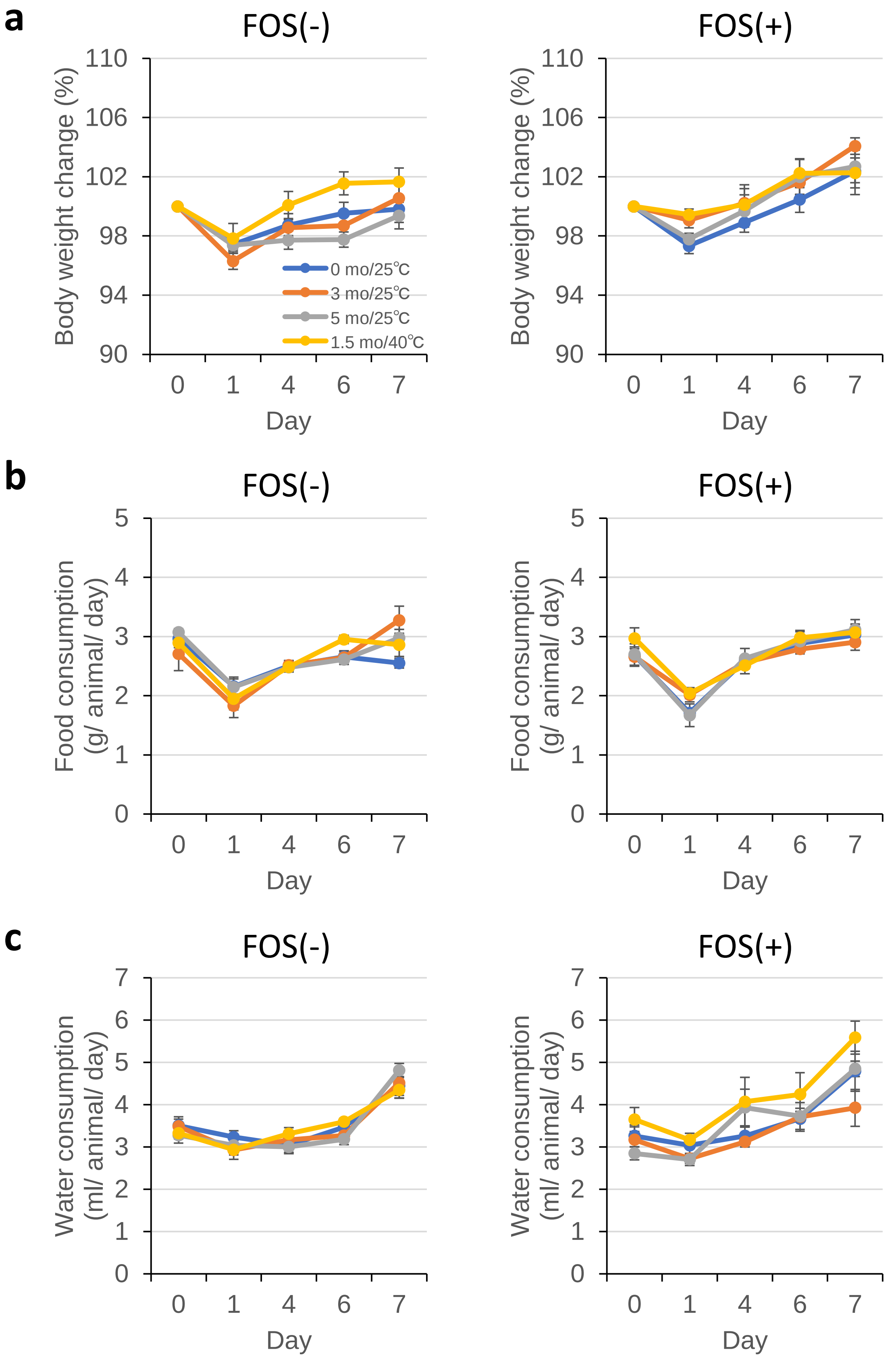

Supplementary Figure 2

Supplementary Table 1 Formula of original and modified AIN-93G (per 100g of diets)

Nutritional composition	Unit	Original AIN-93G	Modified AIN-93G	Modified AIN-93G with FOS
Protein	g	18.07	18.07	18.07
Nitrogen-free extract	g	57.60	57.60	57.60
Fat	g	7.26	7.26	7.26
kcal		368.00	368.00	368.00
Vitamin A	IU	400.0	2000.0	2000.0
Vitamin D3	IU	100.0	500.0	500.0
Vitamin E	mg	7.5	37.5	37.5
Vitamin K1	mg	75.0	375.0	375.0
Vitamin K3	mg	-	-	-
Vitamin B1	mg	0.6	3.0	3.0
Vitamin B2	mg	0.6	3.0	3.0
Vitamin B6	mg	0.7	3.5	3.5
Vitamin B12	mg	2.5	12.5	12.5
Biotin	mg	20.0	100.0	100.0
Folic acid	mg	0.2	1.0	1.0
Pantothenic acid	mg	1.6	8.0	8.0
Nicotinic acid	mg	3.0	15.0	15.0
Choline bitartrate	g	0.25	0.5	0.5
Casein	%	20.00	20.00	20.00
L-Cystine	%	0.30	0.30	0.30
Corn Starch	%	39.75	39.74	39.74
Cellulose	%	5.00	5.00	-
FOS	%	-	-	5.00
Sucrose	%	10.00	10.00	10.00
Soybean oil	%	7.00	7.00	7.00
t-butylhydroquinone	%	0.0014	0.014	0.014
AIN93G-Mineral mix	%	3.50	3.50	3.50
AIN93G-Vitamin mix	%	1.00	-	-
Modified Vitamin mix	%	-	1.00	1.00

Supplementary Table 2 Comparison of vitamin content in mouse diets

Nutritional composition	Unit	Formula of Original AIN-93G	Formula of Modified AIN-93G	Quantitative value of Modified AIN-93G stored for 1year at 25℃	Requirement of the mouse [Ref 11]
Vitamin A	IU	400	2000	1900	240
Vitamin E	mg	7.5	37.5	25.7	2.2
Vitamin B1	mg	0.6	3.0	1.64	0.3
